# Robust Beamforming Based on Covariance Matrix Reconstruction in FDA-MIMO Radar to Suppress Deceptive Jamming

**DOI:** 10.3390/s22041479

**Published:** 2022-02-14

**Authors:** Fuhai Wan, Jingwei Xu, Zhenrong Zhang

**Affiliations:** 1 School of Computer and Electronic Information, Guangxi University, Nanning 530004, China; wanfuhai7@163.com (F.W.); zzr76@gxu.edu.cn (Z.Z.); 2 National Lab of Radar Signal Processing, Xidian University, Xi’an 710071, China

**Keywords:** frequency diverse array (FDA), deceptive jamming suppression, robust beamforming, steering vector mismatch, covariance matrix reconstruction

## Abstract

Frequency diverse array (FDA)-multiple-input multiple-output (MIMO) radars can generate a range-angle two-dimensional transmit steering vector (SV), which is capable of suppressing mainbeam deceptive jamming in the transmit–receive frequency domain by utilizing additional degrees of freedom (DOFs) in the range dimension. However, when there are target SV mismatch, covariance matrix estimation error and target contamination, the jamming suppression performance degrades severely. In this paper, a robust adaptive beamforming algorithm for anti-jammer application based on covariance matrix reconstruction is proposed in FDA-MIMO radar. In this method, the residual noise is further determined by using the spatial power spectrum estimation approach, which results in improved estimation accuracy of the signal covariance matrix and the desired target SV. The jamming SV is obtained from vectors in the intersection of two subspaces (namely, the signal-jamming subspace derived from the sample covariance matrix (SCM) and the jamming subspace generated from the jamming covariance matrix) by an alternating projection algorithm. Furthermore, the jamming power is obtained by exploiting the orthogonality between the different SVs. With the obtained parameters of target and jamming, the optimal adaptive beamformer weight vector is calculated. Simulation results demonstrate that the proposed algorithm can cope with the mainbeam deceptive jamming suppression under various model mismatches and has excellent performance over a wide range of signal-to-noise ratios (SNRs).

## 1. Introduction

With the invention of electronic countermeasures technologies, active deceptive jamming has caused significant repercussions such as impaired information collecting capabilities and resource occupancy in radar systems [1,2]. Jammers equipped with digital radio frequency memory (DRFM) can form active deceptive jamming that is coherent with real target echo by intercepting, sampling, parameter (time delay, Doppler frequency) modulation, and forwarding radar signals, causing the radar system to misidentify false targets as real targets, resulting in the loss of real target and air situation anomalies [3]. In general, there are two types of mainbeam deceptive jamming. The first is jamming that locates inside the main lobe, typical applications such as pods and decoys, which protect the target by forwarding intercepted radar signals that form a coherent scattering source with the signals dispersed directly by the target. The second category with identical angle of the target, mainly generated by self-defense jammers carried by the target itself [4]. Since the characteristics of such mainbeam deceptive jamming and target signal in frequency, temporal, spatial and polarization domains are essentially the same, conventional radar with corresponding algorithms become invalid in anti-jamming, calling for novel scheme of radar and signal processing algorithms.

The frequency diversity array (FDA)-multiple-input multiple-output (MIMO) radar introduces a frequency step amount far smaller than the reference carrier frequency between the transmitting array elements, and uses MIMO technology to separate the transmit waveform at the receiver, resulting in a range-angle-dependent transmit steering vector (SV) [5]. Range-dependent mainbeam deceptive jamming cancellation can be achieved by using the increased range dimension controllable degrees of freedom (DOFs). Recently, the countermeasures of mainbeam deceptive jamming based on the novel FDA-MIMO radar system have attracted tremendous attention [6,7,8,9,10,11,12,13].

As stated in [6], two-dimensional adaptive beamforming is adopted in FDA-MIMO radar to suppress mainbeam deceptive jamming. However, the model in [6] ignores the stored modulation time of the jamming signal in the jammer and assumes that all false targets generated by the same jammer have the same transmit SVs, which is only a specific situation in practice. In [7], deceptive jamming can be effectively mitigated with high probability by combining a cone-domain detector and a dual-pulse detection strategy in FDA-MIMO radar. A non-uniform sample detection approach is proposed to enhance the jamming suppression effectiveness under non-perfect orthogonal waveform situations in [8]. Sample selection employing subspace projection and signal power detection can enhance the capacity of jamming covariance matrix estimation and achieve effective jamming mitigation [9]. In [10], a “low-rank + low-rank + sparse” decomposition technique is utilized to extract the low-rank order desired signal and restrain deceptive jamming. Optimizing the frequency step between each array element using the simulated annealing algorithm [11] can also accomplish adaptive anti-mainbeam deceptive jamming. In [12], a preset-nulling broadening beamformer is proposed to address the jamming suppression problem when spatial frequency mismatch exists. For non-uniformly spaced FDA radars, a cognitive adaptive anti-jamming method based on phase center is suggested [13].

However, the adaptive approaches employed to combat mainbeam deceptive jamming in [6,8,9,11,12] are extremely susceptible to signal SV mismatches induced by observed direction error [14], array geometry error [15], channel gain and phase uncertainty [16], and incoherent local scattering [17]. Furthermore, when the training sample is contaminated by the desired signal, the aforementioned jamming suppression techniques will suffer performance degradation, resulting in the “self-cancellation” of the desired signal. Therefore, robust adaptive beamforming (RAB) in FDA-MIMO radar are required to cope with various model mismatches and strengthen mainbeam deceptive jamming suppression capability.

Various RAB strategies have been presented to enhance the robustness against the model mismatch. These RAB approaches can be roughly classified into four categories: diagonal loading (DL) [18,19,20,21], eigenspace projection [22,23,24], uncertainty set constraints, and interference-plus-noise covariance matrix (IPNCM) reconstruction. DL belongs to one of the most classical RAB techniques, which is implemented by regularizing the Capon minimum variance problem. However, the drawback of this method is that it is difficult to determine the appropriate diagonal loading level for different application scenarios. Although parameter-free solutions for automatically evaluating the loading level have been proposed in [20,21], they perform poorly when the SV mismatch is severe and the input signal-to-noise ratio (SNR) grows. Uncertainty set constraint techniques, such as worst-case performance-optimized (WCPO) beamformer [25,26,27], doubly constrained robust Capon beamformer [28], probabilistically constrained beamformer [29], and linear programming beamformer [30], estimate the desired signal SV by setting a spherical or elliptical uncertainty set associated with the desired signal SV. However, these constrained beamformers often employ the sample covariance matrix (SCM) rather than the real IPNCM, so the jamming rejection performance deteriorates when the desired signal exists with high SNR.

To eliminate the redundant desired signal component in the SCM, RAB based on IPNCM reconstruction has been proposed recently. Gu et al. [31] designed a RAB algorithm based on IPNCM reconstruction and desired signal SV estimation, in which the IPNCM is reconstructed by integrating the Capon spectrum over an angular domain that separates the desired target, whereas the desired signal SV is estimated by solving a quadratic constrained quadratic programming (QCQP) problem. This algorithm has remarkable performance in the occasion of small samples and large look observation error, with the drawback that integration leads to heavy computation and difficulty in coping with array geometry error. In [32], the robust Capon beamformer is suggested to estimate the jamming SV eventually reconstructing the IPNCM, it can address the array geometry error to some extent. The method in [33] reconstructs the IPNCM using the annulus uncertainty set associated with the jamming SV, which adequately resolves all remaining mismatch phenomena except channel gain and phase error, but the integral interval is difficult to determine. While [34] can effectively cope with large look direction errors, but achieving optimal performance necessitates that the incidence directions of the desired target and jamming be sufficiently separated, and the SNR is not close to the interference-to-noise ratio (INR). To reduce the computational complexity of the IPNCM, a RAB method based on spatial power spectrum sampling and matrix tapered technique is devised in [35], but more array elements are required to ensure excellent performance. Zhang et al. [36] creatively proposed an improved factor for assessing the effectiveness of reconstruction and determining whether to reconstruct the IPNCM. A blocking matrix containing the presumed desired signal SV and a small power adjustment factor is applied to remove the desired signal from the SCM in [37] for further improving the beamformer robustness. Despite the low algorithm complexity, satisfying the intended signal blocking characteristic under high SNR is difficult. In [38], it is firstly verified that accurate estimation of jamming power has minimal performance improvement for reconstruction-based beamformers, and a RAB with simplified jamming power estimation is provided. In [39], the signal subspace projection is established to calculate the target and jamming power, and a RAB is devised based on these power estimates. As the accuracy of the Capon spatial spectrum in [31] deteriorates dramatically when coherent signals exist, a low complexity RAB based on the maximum entropy power spectrum is introduced in [40], but it ignores the jamming SV distortion when dealing with arbitrary mismatches. In [41], a novel desired signal SV optimization approach is proposed by designing a desired signal power estimator with uncertainty set constraints, which is more robust to look observation error and array geometry error. The above-mentioned RAB involves three strategies in reconstructing the IPNCM. The first is to remove the desired signal from the SCM [37,41]. The second approach is to reconstruct the IPNCM by integrating the Capon power spectrum over an angular domain containing the jamming [31,33,36]. The third method constructs the IPNCM by estimating the entire jamming SVs and power, as well as the noise term [32,38,39,40]. Obviously, the third method outperforms the former two in terms of reconstruction accuracy. The proposed RAB in this paper based on FDA-MIMO radar falls in the third category.

In this paper, the feasibility of FDA-MIMO radar against mainbeam deceptive jamming is investigated. To improve the anti-jamming performance in the presence of desired signal SV mismatches and IPNCM estimation error, a RAB method that eliminates residual noise to estimate the desired signal SV and reconstructs the IPNCM using subspace projection is proposed to suppress the jamming. Assuming that the prior knowledge of regions where the desired target and jamming locates is known. Firstly, the modified Capon spatial spectrum with residual noise removed is integrated over the desired target region to reconstruct the signal covariance matrix. To accurately estimate the desired signal SV, the reconstructed matrix is eigen-decomposed and the principal eigenvalues are utilized. Secondly, the signal-jamming subspace is determined by eigen-decomposing the SCM, then integrating the modified Capon spatial spectrum over the jamming domain to reconstruct the jamming covariance matrix, which is eigen-decomposed to yield the jamming subspace. With the alternating projection algorithm, the jamming SV can be precisely approximated from the vectors in the intersection of these two subspaces. Furthermore, orthogonality between the different SVs can be used to deduce jamming power. Based on the aforementioned accurate estimation, the reconstruction performance of IPNCM can be enhanced. Finally, the optimal weight vector of the proposed RAB algorithm is determined. Simulation results demonstrate that the proposed beamformer can effectively cope with various model mismatches when suppressing the mainbeam deceptive jamming, and its performance in robustness and output is superior to many existing reconstruction-based beamformers.

The rest of this paper is organized as follows. In Section 2, the signal model of deceptive jamming in FDA-MIMO radar is established. A robust beamforming jamming suppression approach based on FDA-MIMO radar is proposed in Section 3. Simulation results and performance analyses are provided in Section 4. Conclusions are summarized in Section 5.

## 2. FDA-MIMO Radar Signal Model

### 2.1. Desired Signal Model

Without loss of generality, we consider a colocated FDA-MIMO radar consisting of *M* transmit elements and *N* receive elements, both of which are uniform linear arrays. With the first element as a reference, the linear frequency increment Δf is applied in the transmit array. Thus, the transmit frequency corresponding to the *m*-th element is

(1) $$f_{m}=f_{0}+\left(m-1\right)\Delta f,m=1,2,\ldots ,M$$

where $f_{0}$ is the reference carrier frequency. The transmitted signal of the *m*-th element is expressed as

(2) $$x_{m}\left(t\right)=\mathrm{rect}\left(\frac{t}{T_{p}}\right)\psi_{m}\left(t\right)e^{j2\pi f_{m}t}$$

where $\mathrm{rect}\left(\frac{t}{T_{p}}\right)\;=\;\left\{\begin{array}{c} 1\;\;\;\;\;\;\;\;\;\;\;\;0\leq t\leq T_{p}\\ 0\;\;\;\;\;\;\;\;\;\;\;else \end{array}\right.$ denotes the pulse modulation function, $T_{p}$ represents the pulse duration and $\psi_{m}\left(t\right)$ is the baseband envelope. Consider a point target at $\left(r_{s},\theta_{s}\right)$ in the far-field under the narrowband assumption, the backscattered signal from this target received by the *n*-th element can be approximately written as

(3) $$\begin{array}{c} y_{s,n}\left(t-\tau_{s}\right)\approx \sum_{m=1}^{M}\rho_{s0}\cdot \mathrm{rect}\left(\frac{t-\tau_{s}}{T_{p}}\right)\cdot \psi_{m}\left(t-\tau_{s}\right)\cdot e^{j2\pi f_{0}\left(t-\tau_{mn}\right)}\cdot e^{j2\pi \left(m-1\right)\Delta f\left(t-\tau_{mn}\right)} \end{array}$$

where $\rho_{s0}$ denotes the complex-valued target coefficient, which includes antenna transmitting and receiving, electromagnetic wave propagation, backscattering, etc. $\tau_{s}=2r_{s}/c$ is the reference time, $\tau_{mn}=\left[2r_{s}-\left(n-1\right)d_{R}\sin\left(\theta_{s}\right)-\left(m-1\right)d_{T}\sin\left(\theta_{s}\right)\right]/c$ represents time delay between the *m*-th transmit and the *n*-th receive element. $d_{R}$ and $d_{T}$ are the receive and transmit element spacings, respectively. *c* is the speed of light. After analog mixing of the reference carrier frequency for the *m*-th transmitted signal received from the *n*-th channel, digital mixing related to the frequency step and matched filtering, the following signal processing results can be derived

(4) $$\begin{array}{c} y_{mn}\left(t-\tau_{s}\right)=\;\rho_{s}\cdot e^{-j2\pi \left(m-1\right)\Delta f\tau_{s}}\cdot e^{j2\pi \frac{\left(m-1\right)d_{T}\sin\left(\theta_{s}\right)}{\lambda }}\cdot e^{j2\pi \frac{\left(n-1\right)d_{R}\sin\left(\theta_{s}\right)}{\lambda }} \end{array}$$

where $\rho_{s}=\exp\left\{-j2\pi f_{0}\tau_{s}\right\}\cdot \sin c\left(t-\tau_{s}\right)$ is the echo complex-valued coefficient after taking the pulse compression processing gain into account. $\lambda \;=\;c/f_{0}$ denotes the carrier wavelength. Therefore, in vector form, the received target signal is

(5) $$\begin{array}{c} Y_{S}={\left[y_{11}\left(t-\tau_{s}\right),y_{12}\left(t-\tau_{s}\right),\cdots ,y_{MN}\left(t-\tau_{s}\right)\right]}^{T}=\rho_{s}a_{T}\left(r_{s},\theta_{s}\right)⊗a_{R}\left(\theta_{s}\right) \end{array}$$

where ${\left[\cdot \right]}^{T}$ presents transpose operation, ⊗ denotes Kronecker product. $Y_{S}\in C^{MN\times 1}$. $a_{T}\left(r_{s},\theta_{s}\right)\in C^{M\times 1}$ and $a_{R}\left(\theta_{s}\right)\in C^{N\times 1}$ are the transmit and receive SVs of the desired signal, respectively, which have the following general form

(6) $$\begin{array}{cc} a_{T}\left(r_{s},\theta_{s}\right) & =a_{T,r}\left(r_{s}\right)⊙a_{T,\theta }\left(\theta_{s}\right)\;\\ \; & ={\left[1,e^{-j2\pi \Delta f\frac{2r_{s}}{c}},\cdots ,e^{-j2\pi \left(M-1\right)\Delta f\frac{2r_{s}}{c}}\right]}^{T}\\ \; & \;⊙{\left[1,e^{j2\pi \frac{d_{T}}{\lambda }\sin\left(\theta_{s}\right)},\cdots ,e^{j2\pi \left(M-1\right)\frac{d_{T}}{\lambda }\sin\left(\theta_{s}\right)}\right]}^{T} \end{array}$$

(7) $$a_{R}\left(\theta_{s}\right)={\left[1,e^{j2\pi \frac{d_{R}}{\lambda }\sin\left(\theta_{s}\right)},\cdots ,e^{j2\pi \left(N-1\right)\frac{d_{R}}{\lambda }\sin\left(\theta_{s}\right)}\right]}^{T}$$

where ⊙ denotes the Hadamard product. $a_{T,r}\left(r_{s}\right)\in C^{M\times 1}$ is the transmit range SV and $a_{T,\theta }\left(\theta_{s}\right)\in C^{M\times 1}$ is the transmit angle SV. From (6) and (7) that the transmit and receive spatial frequencies of the desired target in FDA-MIMO are as follows

(8) $$f_{T,s}=f_{T,s,r}+f_{T,s,\theta }=-\Delta f\frac{2r_{s}}{c}+\frac{d_{T}}{\lambda }\sin\left(\theta_{s}\right)$$

(9) $$f_{R,s}=f_{R,s,\theta }=\frac{d_{R}}{\lambda }\sin\left(\theta_{s}\right)$$

### 2.2. Jamming Signal Model

**Figure 1:**
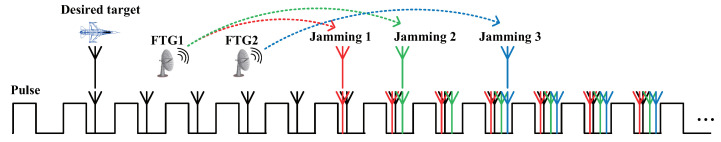
Schematic diagram of generating pseudo-randomly distributed range deceptive jamming.

Considering that *J* false target generators (FTGs) in the far-field where the *j*-th FTG is located at $\left(r_{j},\theta_{j}\right)$. The intercepted radar signal is delayed and forwarded by the FTGs, which form *K* deceptive jamming with pseudo-random distribution in the fast-time dimension. Figure 1 depicts a simplified diagram of this procedure.

For the *k*-th jamming target generated by the *j*-th FTG, jamming signal corresponding to the *m*-th transmit and the *n*-th receive elements after matching filter can be written as

(10) $$J_{k,mn}\left(t-\tau_{jk}\right)\approx \;\rho_{jk}\cdot e^{-j2\pi \left(m-1\right)\Delta f\tau_{jk}}\cdot e^{j2\pi \left(m-1\right)\frac{d_{T}}{\lambda }\sin\left(\theta_{j}\right)}\cdot e^{j2\pi \left(n-1\right)\frac{d_{R}}{\lambda }\sin\left(\theta_{j}\right)}$$

where $\rho_{jk}=\exp\left\{-j2\pi f_{0}\left(2r_{j}/c+\Delta \tau_{jk}\right)\right\}\cdot \sin c\left[t-\left(2r_{j}/c+\Delta \tau_{jk}\right)\right]$ is the complex-valued scattering coefficient of the *k*-th jamming. $\tau_{jk}\;=\;2r_{j}/c+\Delta \tau_{jk}$ donates the reference delay of the *k*-th jamming. $\Delta \tau_{jk}$ represents the modulation time required for the *j*-th FTG to generate the *k*-th jamming. $\tau_{jk}$ can be considered as the time delay corresponding to the jamming target with equivalent range $r_{jk}$, namely $\tau_{jk}\;=\;2r_{jk}/c$. The *k*-th jamming generated by the *j*-th FTG is arranged in vector form as

(11) $$\begin{array}{c} Y_{JK}={\left[J_{k,11}\left(t-\tau_{jk}\right),J_{k,12}\left(t-\tau_{jk}\right),\cdots ,J_{k,MN}\left(t-\tau_{jk}\right)\right]}^{T}=\rho_{jk}a_{T}\left(r_{jk},\theta_{j}\right)⊗a_{R}\left(\theta_{j}\right) \end{array}$$

where $Y_{JK}\in C^{MN\times 1}$. $a_{T}\left(r_{jk},\theta_{j}\right)\in C^{M\times 1}$ and $a_{R}\left(\theta_{j}\right)\in C^{N\times 1}$ denote the transmit and receive SVs of jamming, respectively, which are the same in form as (6) and (7), but different in range and angle. Similarly, the transmit and receive spatial frequencies corresponding to the *k*-th jamming forwarded by the *j*-th jammer are as follows:

(12) $$\begin{array}{c} f_{T,j,k}=f_{T,jk,r}+f_{T,jk,\theta }=-\Delta f\frac{2r_{jk}}{c}+\frac{d_{T}}{\lambda }\sin\left(\theta_{j}\right) \end{array}$$

(13) $$\begin{array}{c} f_{R,j,k}=f_{R,jk,\theta }=\frac{d_{R}}{\lambda }\sin\left(\theta_{j}\right) \end{array}$$

### 2.3. Receiving Signal Model

Considering the desired signal, deceptive jamming as well as Gaussian noise, the total received FDA-MIMO radar snapshots take the form

(14) $$\begin{array}{cc} Y(t) & =Y_{S}\left(t\right)+Y_{JK}\left(t\right)+N\left(t\right)\\ \; & =\rho_{s}\left(t\right)a_{T}\left(r_{s},\theta_{s}\right)⊗a_{R}\left(\theta_{s}\right)+\sum_{j=1}^{J}\sum_{k=1}^{K}\rho_{jk}\left(t\right)a_{T}\left(r_{jk},\theta_{j}\right)⊗a_{R}\left(\theta_{j}\right)+N\left(t\right) \end{array}$$

where $\rho_{s}\left(t\right)\;=\;\rho_{s}\cdot \delta \left(t-\tau_{s}\right)$ represents the range bin of the desired target corresponding to time delay $\tau_{s}$. $\rho_{jk}\left(t\right)\;=\;\rho_{jk}\cdot \delta \left(t-\tau_{jk}\right)$ denotes that the *k*-th deceptive jamming generated by the *j*-th FTG dwelling at the range bin corresponding to time delay $\tau_{jk}$.

**Figure 2:**
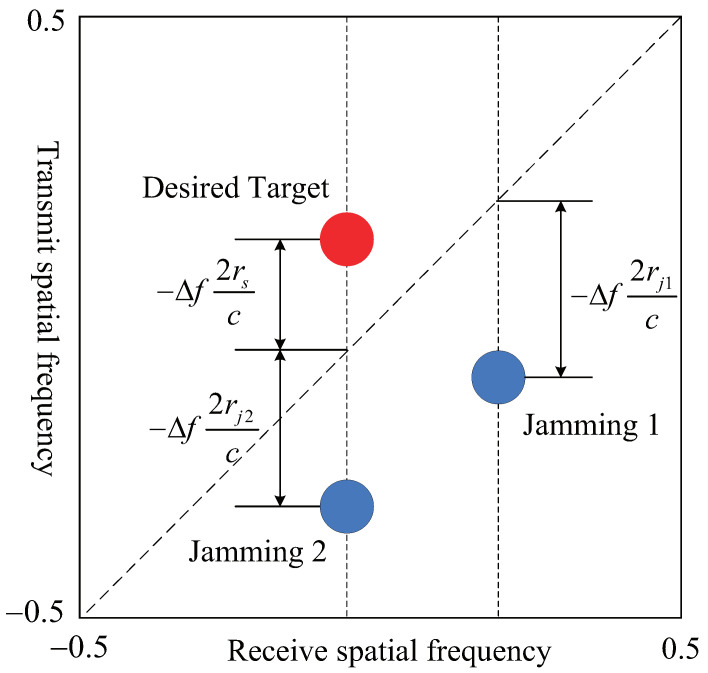
Schematic diagram of the power spectrum distribution of the desired target and jamming.

Comparing (8), (9), (12) and (13), the following conclusions can be drawn:

1. If the desired target and jamming spatial angle differ, i.e., $\theta_{s}\neq \theta_{j}$, then they can be distinguished directly in FDA-MIMO radar by employing the receive spatial frequency dimension.
2. If the angle between the desired target and jamming is nearly the same, that is $\theta_{s}\approx \theta_{j}$, they cannot be divided by using the receive spatial frequency. Nevertheless, the principal range of the desired target is $r_{s}$, whereas jamming is generated by the FTG via time delay modulation and has an equivalent range of $r_{jk}$. As a result, they differ in range dimension. It is considered to discern between the desired target and jamming in the transmit–receive frequency domain. Figure 2 illustrates the power spectrum distribution diagram of the desired target and jamming in the transmit-receive frequency domain of the FDA-MIMO radar.

## 3. Robust Decepticve Jamming Suppression

### 3.1. Background

After the desired target and deceptive jamming generated by delay forwarding are separated in the transmit–receive frequency domain of the FDA-MIMO radar due to the range disparity, we can utilize two-dimensional RAB techniques to eliminate the range mismatch jamming. Generally, the optimal weight vector w is determined by maximizing the output signal-to-interference-plus-noise ratio (SINR), which is depicted below

(15) $$\mathrm{SINR}\;=\;\frac{\sigma_{s}^{2}{\left|w^{H}\tilde{a}\left({\tilde{r}}_{s},{\tilde{\theta }}_{s}\right)\right|}^{2}}{w^{H}R_{j+n}w}$$

where $\sigma_{s}^{2}$ denotes the desired signal power. $\tilde{a}\left({\tilde{r}}_{s},{\tilde{\theta }}_{s}\right)\;=\;{\tilde{a}}_{T}\left({\tilde{r}}_{s},{\tilde{\theta }}_{s}\right)⊗{\tilde{a}}_{R}\left({\tilde{\theta }}_{s}\right)$ is the presumed desired SV of the desired target with ${\tilde{r}}_{s}$ and ${\tilde{\theta }}_{s}$ standing for the presumed range and angle. $R_{j+n}$ represents the theoretical IPNCM, which is expressed as

(16) $$\begin{array}{cc} R_{j+n} & =E\left\{\left(Y_{J}\left(t\right)+N\left(t\right)\right){\left(Y_{J}\left(t\right)+N\left(t\right)\right)}^{H}\right\}\\ \; & =R_{j}+\sigma_{n}^{2}I_{MN} \end{array}$$

where $R_{j}$ is the real jamming covariance matrix, $\sigma_{n}^{2}$ represents the Gaussian white noise power, and $I_{MN}\in C^{MN\times MN}$ denotes the identity matrix. Maximizing the output SINR can be equivalent to the following optimization problem

(17) $$\begin{array}{cc} \; & \min_{w}w^{H}R_{j+n}w\\ \; & s.t.\;\;\;\;\;\;\;\;w^{H}\tilde{a}\left({\tilde{r}}_{s},{\tilde{\theta }}_{s}\right)=1 \end{array}$$

Which yield the minimum variance distortionless response (MVDR) beamformer

(18) $$\begin{array}{c} w_{opt}=\frac{R_{j+n}^{-1}\tilde{a}\left({\tilde{r}}_{s},{\tilde{\theta }}_{s}\right)}{{\tilde{a}}^{H}\left({\tilde{r}}_{s},{\tilde{\theta }}_{s}\right)R_{j+n}^{-1}\tilde{a}\left({\tilde{r}}_{s},{\tilde{\theta }}_{s}\right)} \end{array}$$

MVDR is widely employed by FDA-MIMO radar owing to its fast convergence speed and simple implementation. In practical scenarios, the accurate desired target SV and IPNCM are unavailable attributed to the presence of imprecise target parameter estimation, fewer snapshots, array geometry error, array channel inconsistency, and incoherent local scattering. The anti-jamming effectiveness of the MVDR beamformer in these instances deteriorates considerably. Following that, the IPNCM error and the desired target range-angle SV error are introduced individually.

The SV error will occur when inaccurate target range-angle information is applied to estimate the desired target SV. Therefore, in the presence of range and angle mismatch, the precise desired target SV can be stated as

(19) $$\begin{array}{c} a\left(r_{s},\theta_{s}\right)\;=\;\tilde{a}\left({\tilde{r}}_{s},{\tilde{\theta }}_{s}\right)+\delta \end{array}$$

where δ denotes the desired target SV error. When training samples are contaminated with the desired target, the IPNCM error appears. Generally, the SCM $\hat{R}$ obtained from *L* training samples is utilized to approximate the IPNCM $R_{j+n}$.

(20) $$\begin{array}{c} \hat{R}\overset{\Delta }{\;=}\frac{1}{L}\sum_{l=1}^{L}Y\left(l\right)Y^{H}\left(l\right) \end{array}$$

where Y(l) is the training samples received by the array in the test region. When some training samples contain the desired signal, the SCM can be further written as

(21) $$\begin{array}{c} \hat{R}\;=\;{\tilde{R}}_{j+n}+{\hat{R}}_{error} \end{array}$$

where ${\hat{R}}_{error}$ is the covariance matrix estimation error due to contaminated training samples and ${\tilde{R}}_{j+n}$ is the estimated IPNCM. Obviously, ${\tilde{R}}_{j+n}$ is the ideal estimate of the theoretical $R_{j+n}$, whereas ${\hat{R}}_{error}$ is considered as the undesired term. Therefore, it is vital to concentrate on robust adaptive beamforming methods, which are insensitive to model mismatches to suppress jamming.

### 3.2. Proposed Method

In this section, we proposed a deceptive jamming-resistant adaptive beamforming approach based on FDA-MIMO radar, which eliminates the adverse impacts of range-angle mismatch and IPNCM error. This method will correct the desired target SV and reconstruct the IPNCM, including three steps, i.e., (1) Integrating the Capon spatial spectrum with residual noise removed over the target range-angle domain to estimate the desired target SV. (2) Estimating all jamming SVs utilizing the alternating projection algorithm. (3) Calculating the jamming power by exploiting the orthogonality between the different SVs and finally reconstructing the IPNCM.

Step 1: Residual noise analysis and desired signal SV estimation

**Figure 3:**
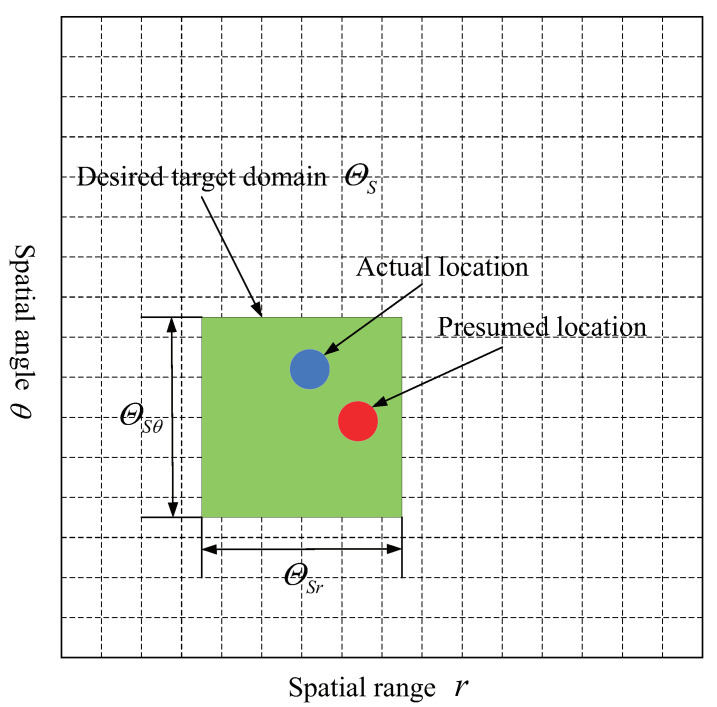
Illustration of Desired target domain in range-angle whole domain.

Assume that the target region $\Theta_{S}$ in the whole range-angle domain, where the possible spatial range and angle of target locates, is known in prior, as depicted in Figure 3. The presumed target position inside the target domain is inconsistent with the actual target position, that is, there is a mismatch between the presumed target spatial range and angle and the actual target range and angle. Define the interval of possible target spatial range *r* and angle θ as $\Theta_{Sr}$ and $\Theta_{S\theta }$ respectively, which can be determined using range measurement methods and DOA estimation techniques, then $\Theta_{S}$ can be denoted as $\Theta_{S}=[\Theta_{Sr},\Theta_{S\theta }]$.

Many existing approaches for estimating the desired target SV based on covariance matrix reconstruction are implemented by integrating the Capon spatial spectrum over the region $\Theta_{S}$. The reason for utilizing Capon spatial spectrum is that it has excellent resolution properties, lacks side lobes and can accurately reflect the actual jamming distribution. The Capon power spectrum in FDA-MIMO radar takes the following form:

(22) $$\begin{array}{c} \hat{P}\left(r,\theta \right)\;=\;\frac{1}{a^{H}\left(r,\theta \right){\hat{R}}^{-1}a\left(r,\theta \right)} \end{array}$$

where $a^{H}\left(r,\theta \right)\;=\;a_{T}\left(r,\theta \right)⊗a_{R}\left(r,\theta \right)$ denotes the SV in region $\Theta_{S}$. The desired target covariance matrix based on (22) is

(23) $$\begin{array}{cc} {\hat{R}}_{S} & =\underset{\Theta_{S}}{\;\int \int \;}\hat{P}\left(r,\theta \right)a\left(r,\theta \right)a^{H}\left(r,\theta \right)drd\theta \\ \; & =\underset{\Theta_{S}}{\;\int \int \;}\frac{a\left(r,\theta \right)a^{H}\left(r,\theta \right)}{a^{H}\left(r,\theta \right){\hat{R}}^{-1}a\left(r,\theta \right)}drd\theta \end{array}$$

However, the existing target SV estimation strategies based on (23) seldom consider the influence of the residual noise in the Capon spatial spectrum estimators, which can lead to an inaccurate reconstructed covariance matrix. The existence of residual noise can be verified as follows. Assuming that the received signal only comprises complex Gaussian white noise, the covariance matrix satisfies $R\;=\;\sigma_{n}^{2}I_{MN}$ and the output after utilizing the Capon spatial spectrum is

(24) $$\begin{array}{cc} P_{Residual}\left(r,\theta \right) & =\frac{1}{a^{H}\left(r,\theta \right){\left(\sigma_{n}^{2}I_{MN}\right)}^{-1}a\left(r,\theta \right)}\\ \; & =\frac{\sigma_{n}^{2}}{MN} \end{array}$$

(24) demonstrates that the residual noise power is 1/(MN) of the actual noise power. Further, supposing a desired target (or jamming) at spatial coordinate $\left(r_{d},\theta_{d}\right)$, the covariance matrix constructed from the echo data is $R_{d+n}\;=\;{\sigma_{d}}^{2}a\left(r_{d},\theta_{d}\right)a^{H}\left(r_{d},\theta_{d}\right)+\sigma_{n}^{2}I_{MN}$, where ${\sigma_{d}}^{2}$ represents the desired target (or jamming) power. Correspondingly, (24) is modified to

(25) $$\begin{array}{cc} P(r_{d},\theta_{d}) & =\frac{1}{a{\left(r_{d},\theta_{d}\right)}^{H}{\left(R_{d+n}\right)}^{-1}a\left(r_{d},\theta_{d}\right)}\\ \; & ={\sigma_{d}}^{2}+\frac{\sigma_{n}^{2}}{MN} \end{array}$$

**Figure 4:**
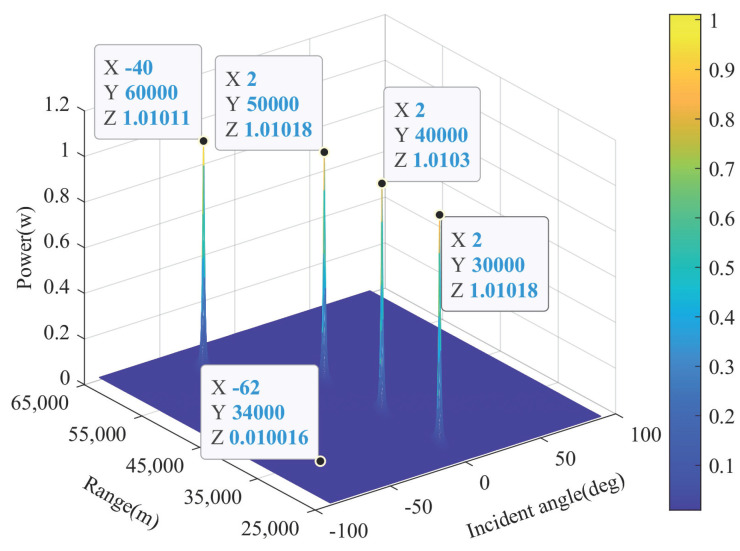
Power spectrum distribution of desired signal and jamming.

(25) illustrates that the power estimation at the desired target spatial position contains signal and residual noise. Figure 4 exhibitions the power amplitude in whole range-angle space according to (22), where the receiving and transmitting elements of FDA-MIMO radar are M=N=10, the power of one desired target at $\left(2^{\circ },30\;\mathrm{km}\right)$ and three jamming at $\left(2^{\circ },40\;\mathrm{km}\right)$, $\left(2^{\circ },50\;\mathrm{km}\right)$ and $\left(-40^{\circ },60\;\mathrm{km}\right)$ are set as 1w, respectively, and complex Gaussian white noise with variance $\sigma_{n}^{2}\;=\;1$ is employed. It is observed that the power fluctuates around 0.01w in the range-angle domain away from the spectral peak, confirming (24). Furthermore, the power at four spectral peaks is roughly 1.01 w, which exceeds their actual value of 1 w and is consistent with (25).

Although the power of residual noise $\sigma_{n}^{2}/MN$ is relatively small in comparision to the desired signal, the integration operation is utilized when reconstructing the signal covariance matrix, which obtains much redundant information related to residual noise, resulting in an inaccurate reconstruction matrix, especially in low SNR scenarios. Therefore, the residual noise power magnitude is evaluated first. The desired target domain $\Theta_{S}$, which is a priori knowledge, has been defined before. Similarly, the union of multiple range-angle regions where jamming may be dispersed is signified the jamming domain $\Theta_{J}$, i.e., $\Theta_{J}=\Theta_{J-1}\cup \Theta_{J-2}\cup \cdots \cup \Theta_{J-K}$, *K* denotes the total number of jamming. Subsequently, the complement region of the target and jamming domains is assigned as the noise domain $\Theta_{N}$. As a result, the entire range-angle domain Θ can be constituted of $\Theta =\Theta_{S}\cup \Theta_{J}\cup \Theta_{N}$. We consider sampling $\Theta_{N}$ uniformly and then adopting the Capon spectrum estimator to estimate the residual noise power ${\hat{\sigma }}_{n}^{2}$ as follows:

(26) $$\begin{array}{c} {\hat{\sigma }}_{n}^{2}=\frac{1}{Q}\sum_{q=1}^{Q}\frac{1}{a^{H}\left(r_{q},\theta_{q}\right){\hat{R}}^{-1}a\left(r_{q},\theta_{q}\right)},\left(r_{q},\theta_{q}\right)\in \Theta_{N} \end{array}$$

where *Q* denotes the number of samples and $\left(r_{q},\theta_{q}\right)$ is the discrete sample value within $\Theta_{N}$. Unlike (23), we eliminate residual noise from the Capon spatial spectrum in this paper, and $\hat{P}\left(\theta_{p}\right)-{\hat{\sigma }}_{n}^{2}$ indicates a more accurate signal power distribution inside $\Theta_{S}$, which can be employed to more accurately reconstruct the desired target covariance matrix as follows

(27) $$\begin{array}{c} {\hat{R}}_{S}=\underset{\Theta_{S}}{\;\int \int \;}\left[\hat{P}\left(r,\theta \right)-{\hat{\sigma }}_{n}^{2}\right]a\left(r,\theta \right)a^{H}\left(r,\theta \right)drd\theta \end{array}$$

As shown in Figure 3, in order to efficiently compute (27), the whole range-angle plane is discretized into $P\;=\;P_{r}P_{s}$ grid points, where $P_{r}$ is the range dimension sampling number and $P_{\theta }$ is the angle dimension sampling number. Each grid point represents a range-angle SV $a(r_{p},\theta_{p}),p=1,2,\cdots ,P$. Thus, the set of range-angle SVs located in $\Theta_{S}$ can be denoted as $\left\{a\left(r_{1},\theta_{1}\right),a\left(r_{2},\theta_{2}\right),\cdots ,a\left(r_{P_{0}},\theta_{P_{0}}\right)\right\}$, where $P_{0}\;=\;P_{r0}P_{\theta 0}$ is the number of range-angle SVs in $\Theta_{S}$, and $P_{r0}$ and $P_{\theta 0}$ represent the sampling number of range dimension and angle dimensional, respectively. After substituting the integral with the discrete point summation, (27) is approximated as

(28) $$\begin{array}{c} {\hat{R}}_{S}\;=\;\sum_{p=1}^{P_{0}}\left[\hat{P}\left(r_{p},\theta_{p}\right)-{\hat{\sigma }}_{n}^{2}\right]a\left(r_{p},\theta_{p}\right)a^{H}\left(r_{p},\theta_{p}\right) \end{array}$$

Consider the eigendecomposition of ${\hat{R}}_{S}$ as follows

(29) $$\begin{array}{c} {\hat{R}}_{S}\;=\;\sum_{i=1}^{MN}\gamma_{i}e_{i}e_{i}^{H} \end{array}$$

where $\gamma_{i},i=1,2,\cdots ,MN$ expresses the eigenvalues of ${\hat{R}}_{S}$ in descending order, i.e., $\gamma_{1}\geq \gamma_{2}\geq \cdots \geq \gamma_{MN}$, and $e_{i}$ is the eigenvector corresponding to the eigenvalue $\gamma_{i}$. In fact, the principal eigenvector $e_{1}$ of ${\hat{R}}_{S}$ covers the most information components of the desired signal, which can be deemed to the estimated desired signal SV

(30) $$\begin{array}{c} {\hat{a}}_{S}=\sqrt{MN}e_{1} \end{array}$$

Step 2: Jamming SV estimation

For the estimation of jamming SV, two jamming-related subspaces are considered. First, the SCM is eigen-decomposed as follows:

(31) $$\begin{array}{c} \hat{R}\;=\;\sum_{i=1}^{MN}\eta_{i}u_{i}u_{i}^{H}=U_{SJ}\Sigma_{SJ}U_{SJ}^{H}+U_{N}\Sigma_{N}U_{N}^{H} \end{array}$$

where $\eta_{i}$ are the eigenvalues of the matrix $\hat{R}$, which are in descending order, i.e., $\eta_{1}\geq \eta_{2}\geq \cdots \geq \eta_{K}>\eta_{K+1}=\cdots =\eta_{MN}=\sigma_{n}^{2}$. The first *K* eigenvalues are related to the desired signal and jamming, and the corresponding eigenvectors are $u_{1},u_{2},\cdots ,u_{K}$, which constitute the signal and jamming subspace $U_{SJ}$. $\Sigma_{SJ}\;=\;diag\left(\eta_{1},\eta_{2},\cdots ,\eta_{K}\right)$ is a diagonal matrix composed of *K* larger eigenvalues. The latter MN−K eigenvalues are completely dependent on noise, which are equal to $\sigma_{n}^{2}$. The eigenvectors corresponding to $\eta_{K+1},\eta_{K+2},\cdots ,\eta_{MN}$ constitute noise subspace $U_{N}$, $\Sigma_{N}\;=\;diag\left(\eta_{K+1},\eta_{K+2},\cdots ,\eta_{MN}\right)$ is a diagonal matrix composed of MN−K smaller eigenvalues. Obviously, the jamming SV depends on the signal plus jamming subspace $U_{SJ}$, so it is considered as the first jamming-related subspace. More specifically, the *k*-th jamming SV lies in the subspace spanned by the column vectors of $U_{SJ}$ as follows:

(32) $$\begin{array}{c} T_{U}=\left\{{\hat{a}}_{k}:{\hat{a}}_{k}\in U_{SJ}\alpha_{SJ}\right\} \end{array}$$

where $\alpha_{SJ}$ is the linear correlation coefficient vector. For the second jamming-related subspace, similar to the idea of reconstructing the desired target covariance matrix in the previous subsection. We discrete the *k*-th jamming domain $\Theta_{J-k}$ into $P_{k}\;=\;P_{rk}P_{\theta k}$ grid points, where $P_{rk}$ and $P_{\theta k}$ represent the sampling numbers of range dimension and angle dimension in $\Theta_{J-k}$, respectively. The *k*-th jamming covariance matrix can be obtained by sampling and summing the Capon power spectrum with the residual noise removed in the range-angle domain where the *k*-th jamming is situated

(33) $$\begin{array}{c} {\hat{R}}_{J-k}\;=\;\sum_{p=1}^{P_{k}}\left[\hat{P}\left(r_{p},\theta_{p}\right)-{\hat{\sigma }}_{n}^{2}\right]a\left(r_{p},\theta_{p}\right)a^{H}\left(r_{p},\theta_{p}\right) \end{array}$$

It should be noted that the number of discretized grid points $P_{k}$ in $\Theta_{J-k}$ is usually equal to the number of discretized grid points $P_{0}$ in $\Theta_{S}$, s.t., $P_{rk}\;=\;P_{r0}$, $P_{\theta k}\;=\;P_{\theta 0}$. The jamming covariance matrix is eigen-decomposed as follows:

(34) $$\begin{array}{c} {\hat{R}}_{J-k}\;=\;\sum_{i=1}^{MN}\mu_{i}v_{i}v_{i}^{H}=V_{J-k}\Upsilon_{J-k}V_{J-k}^{H}+V_{N}\Upsilon_{N}V_{N}^{H} \end{array}$$

where $\mu_{i},i=1,2,\cdots ,MN$ are eigenvalues of ${\hat{R}}_{J-k}$ in descending order and $v_{i}$ is the eigenvector corresponding to the eigenvalue $\mu_{i}$. $\Upsilon_{J-k}\;=\;diag\left(\mu_{1},\mu_{2},\cdots ,\mu_{D}\right)$ is a diagonal matrix composed of *D* larger eigenvalues, and $V_{J-k}\;=\;\left[v_{1},v_{2},\cdots ,v_{D}\right]$ is the *k*-th jamming subspace composed of *D* main eigenvectors. $V_{N}\;=\;\left[v_{D+1},v_{D+2},\cdots ,v_{MN}\right]$ denotes noise subspace, which consists of the eigenvectors corresponding to the remaining MN−D smaller eigenvalues. $\Upsilon_{N}\;=\;diag\left(\mu_{D+1},\mu_{D+2},\cdots ,\mu_{MN}\right)$ is also a diagonal matrix. It should be noted that *D* can be determined by the following constraint relation:

(35) $$\begin{array}{c} \frac{\sum_{i=1}^{D}{\left|\mu_{i}\right|}^{2}}{\sum_{i=1}^{MN}{\left|\mu_{i}\right|}^{2}}\geq \lambda \end{array}$$

where λ is a predetermined constraint factor belonging to interval (0, 1). $V_{J-k}$ is the second subspace associated with jamming. That is to say, the *k*-th jamming SV is also positioned in the space spanned by the columns of $V_{J-k}$ as

(36) $$\begin{array}{c} T_{V-k}=\left\{{\hat{a}}_{k}:{\hat{a}}_{k}\in V_{J-k}\alpha_{J-k}\right\} \end{array}$$

where $\alpha_{J-k}$ is the correlation coefficient vector. As aforementioned, the *k*-th jamming SV can be regarded as inside the intersection $T\overset{\Delta }{\;=}T_{U}\cap T_{V-k}$ of two subspaces. Then, we can adopt the alternating projection algorithm proposed in [42] to handle the subspace intersection. The principle is to construct an iterative relationship for SV utilizing the projection matrix. Consider the jamming model, $a_{k+1}=T_{U}T_{V-k}a_{k}$ can be established, where $T_{U}\;=\;U_{SJ}U_{SJ}^{H}$ and $T_{V-k}=V_{J-k}V_{J-k}^{H}$ represent projection matrices on subspaces $U_{SJ}$ and $V_{J-k}$, respectively. In addition, the presumed jamming SV ${\hat{a}}_{k}\left({\hat{r}}_{k},{\hat{\theta }}_{k}\right)$ can be considered as the initial value of $\left\{a_{k}\right\}$, where $\left({\hat{r}}_{k},{\hat{\theta }}_{k}\right)$ is the presumed incident location. When k→∞, $a_{k+1}$ converges to the actual jamming SV. In other words, when k→∞, $a_{k+1}$ must be composed of the principal eigenvector following eigendecomposition of $T_{U}T_{V-k}$, which contains the most information of $a_{k+1}$, and the maximum eigenvalue of $T_{U}T_{V-k}$ should be equal to 1. The constraint condition is tenable, and the following derivation process is given [43]:

(37) $$\begin{array}{cc} eig_{\max}\left(T_{U}T_{V-k}\right) & \leq eig_{\max}\left(T_{U}\right)\max_{\varpi }\;\varpi^{H}T_{V-k}\varpi \\ \; & =\max_{\underset{\varpi^{H}\varpi =1}{\varpi }\;}\;\frac{\varpi^{H}T_{V-k}\varpi }{\varpi^{H}\varpi }\\ \; & =eig_{\max}\left(T_{V-k}\right)=1 \end{array}$$

where $eig_{\max}\left(\cdot \right)$ is the maximum eigenvalue of a matrix, then the accurate estimation value of the *k*-th jamming SV is expressed as

(38) $$\begin{array}{c} {\hat{a}}_{k}\;=\;\sqrt{MN}P_{r}\left(T_{U}T_{V-k}\right) \end{array}$$

where $P_{r}\left(\cdot \right)$ denotes the principal eigenvector of a matrix. Two strategies are involved to improve the estimation accuracy of jamming SV. The first is the application of vector space projection, which enhances the robustness of the jamming SV error suppression. The second is to eliminate the residual noise and reconstruct the jamming covariance matrix, which also guarantees higher estimation accuracy of the jamming SV to a certain extent.

Step 3: Jamming power estimation and IPNCM reconstruction

The data covariance matrix of received signal Y(t) can be written in the following form:

(39) $$\begin{array}{cc} R & ≜E\left\{Y\left(t\right)Y^{H}\left(t\right)\right\}=AR_{SJ}A+R_{N}\\ \; & =\sum_{k=0}^{K}\sigma_{k}^{2}a\left(r_{k},\theta_{k}\right)a^{H}\left(r_{k},\theta_{k}\right)+\sigma_{n}^{2}I_{MN} \end{array}$$

where $a\left(r_{k},\theta_{k}\right)\;=\;a_{T}\left(r_{k},\theta_{k}\right)⊗a_{R}\left(\theta_{k}\right)$ is the SV of the *k*-th signal (desired target or jamming), A denotes the array manifold, and $R_{SJ}$ and $R_{N}$ are the signal plus jamming covariance matrix and the noise covariance matrix, respectively. The remaining part after removing the noise term from R is expressed as

(40) $$\begin{array}{cc} R_{EN} & =R-R_{N}=R-\sigma_{n}^{2}I_{MN}\\ \; & =\sum_{k=0}^{K}\sigma_{k}^{2}a\left(r_{k},\theta_{k}\right)a^{H}\left(r_{k},\theta_{k}\right) \end{array}$$

Pre-multiplying the above equation by $a^{H}\left(r_{l},\theta_{l}\right)$ of the *l*-th signal and then post-multiplying by $a(r_{l},\theta_{l})$ gives the following result:

(41) $$a^{H}\left(r_{l},\theta_{l}\right)R_{EN}a\left(r_{l},\theta_{l}\right)=a^{H}\left(r_{l},\theta_{l}\right)\left\{\sum_{k=0}^{K}\sigma_{k}^{2}a\left(r_{k},\theta_{k}\right)a^{H}\left(r_{k},\theta_{k}\right)\right\}a\left(r_{l},\theta_{l}\right)$$

The following analysis is performed on (41). If the desired signals or jamming are incident on the array at different ranges and angles, the SVs corresponding to any two signals are approximately orthogonal or completely orthogonal. That is, when k≠l, $a^{H}\left(r_{l},\theta_{l}\right)a\left(r_{k},\theta_{k}\right)\ll a^{H}\left(r_{l},\theta_{l}\right)a\left(r_{l},\theta_{l}\right)$ or $a^{H}\left(r_{l},\theta_{l}\right)a\left(r_{k},\theta_{k}\right)\;=\;0$ exist. Furthermore, $a^{H}\left(r_{l},\theta_{l}\right)a\left(r_{k},\theta_{k}\right)\;=\;MN$ is fulfilled when k=l. The following experiment can demonstrate orthogonal characteristics between different SVs.

(42) $$H=\frac{\left|a^{H}\left(r_{l},\theta_{l}\right)a\left(r_{k},\theta_{k}\right)\right|}{MN}$$

**Figure 5:**
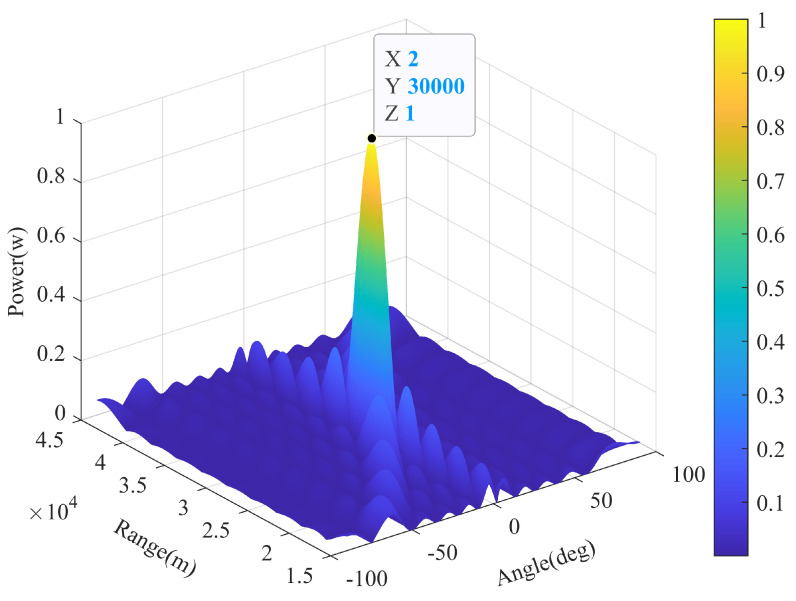
Values of *H* when $\left(r_{k},\theta_{k}\right)$ deviates from $\left(r_{l},\theta_{l}\right)$.

Assume that the number of transmit and receive elements in FDA-MIMO radar matches M=N=10, a signal impinges on the array from $\left(r_{l},\theta_{l}\right)=\left(30\;{\mathrm{km},2}^{\circ }\right)$. Figure 5 depicts the experimental results achieved using (42). It is observed that when $\left(r_{k},\theta_{k})=(r_{l},\theta_{l}\right)$ is satisfied, *H* reaches the maximum value 1, as ${∥a(r_{l},\theta_{l})∥}_{2}\;=\;\sqrt{MN}$. However, when $\left(r_{k},\theta_{k}\right)$ deviates from $\left(r_{l},\theta_{l}\right)$, *H* diminishes a tiny scale, which indicates that the two signal SVs are approximately or completely orthogonal.

Based on the orthogonality between SVs, $\left(r_{k},\theta_{k}\right)$ in (41) is traversed in the whole range-angle domain, and the orthogonal terms are neglected, resulting in

(43) $$\begin{array}{c} a^{H}\left(r_{k},\theta_{k}\right)R_{EN}a\left(r_{k},\theta_{k}\right)=\sigma_{k}^{2}a^{H}\left(r_{k},\theta_{k}\right)a\left(r_{k},\theta_{k}\right)a^{H}\left(r_{k},\theta_{k}\right)a\left(r_{k},\theta_{k}\right) \end{array}$$

$R_{EN}$ is calculated by covariance matrix $\hat{R}$ and noise power ${\hat{\sigma }}_{n}^{2}$, and the jamming SV obtained in the previous paper is substituted into (43), then the power estimation of the *k*-th jamming is written as

(44) $$\begin{array}{c} {\hat{\sigma }}_{k}^{2}=\frac{{\hat{a}}^{H}\left(r_{k},\theta_{k}\right)\left(\hat{R}-{\hat{\sigma }}_{n}^{2}I_{MN}\right)\hat{a}\left(r_{k},\theta_{k}\right)}{{\left|{\hat{a}}^{H}\left(r_{k},\theta_{k}\right)\hat{a}\left(r_{k},\theta_{k}\right)\right|}^{2}} \end{array}$$

Finally, IPNCM can be reconstructed as follows

(45) $$\begin{array}{c} {\hat{R}}_{j+n}\;=\;\sum_{k=1}^{K}{\hat{\sigma }}_{k}^{2}\hat{a}\left(r_{k},\theta_{k}\right){\hat{a}}^{H}\left(r_{k},\theta_{k}\right)+{\hat{\sigma }}_{n}^{2}I_{MN} \end{array}$$

where *K* is the number of deceptive jamming. The proposed weight vector is shown below:

(46) $$\begin{array}{c} {\hat{w}}_{proposed}=\frac{{\hat{R}}_{j+n}^{-1}{\hat{a}}_{S}}{{\hat{a}}_{S}^{H}{\hat{R}}_{j+n}^{-1}{\hat{a}}_{S}} \end{array}$$

The procedures of the proposed robust jamming suppression are summarized in Algorithm 1:
**Algorithm 1:** Proposed RAB Algorithm.1:Calculate the SCM $\hat{R}$ via (20) and estimate the residual noise power ${\hat{\sigma }}_{n}^{2}$ using (26). 2:Reconstruct signal covariance matrix ${\hat{R}}_{S}$ using (28) and desired signal SV ${\hat{a}}_{S}$ (30) is obtained by eigen-decomposing ${\hat{R}}_{S}$. 3:Eigen-decompose $\hat{R}$ to obtain the subspace $U_{SJ}$ (31). Calculate the jamming covariance matrix matrix ${\hat{R}}_{J-k}$ using (33), and eigendecompose ${\hat{R}}_{J-k}$ to get the subspace $V_{J-k}$ (34). 4:Substitute $U_{SJ}$ and $V_{J-k}$ into (38) to estimate the *k*-th jamming SV ${\hat{a}}_{k}$. 5:Calculate jamming power via (44), and ${\hat{R}}_{j+n}$ is reconstructed by (45). 6:Obtain the proposed RAB weight vector ${\hat{w}}_{proposed}$ (46). 7:Distinguish the deceptive jamming in transmit-receive spatial frequency domain by range mismatch. 8:Supress mainbeam deceptive jamming utilizing proposed RAB.

In the proposed algorithm, the computational complexity mainly concentrated on the target SV estimation, the jamming SVs estimation and the IPNCM inversion:

1. The complexity of the target SV estimation can be divided into two componets. The first is to constructe target covariance matrix at the cost of $O({\left(MN\right)}^{2}P_{0})$. The second is to decompose the target covariance matrix costing $O({\left(MN\right)}^{3})$. Therefore, the complexity of solving the target SV estimation is $O(\max\left({\left(MN\right)}^{2}P_{0},{\left(MN\right)}^{3}\right))$.
2. The complexity of the jamming SVs estimation includes three parts. First, it has a complexity of $O({\left(MN\right)}^{3})$ to eigendecomposition of the SCM $\hat{R}$ to obtain $U_{SJ}$. Second, it has complexity of $O(\max\left({\left(MN\right)}^{2}P_{k},{\left(MN\right)}^{3}\right))$ through reconstructing the *k*-th jamming covariance matrix ${\hat{R}}_{J-k}$ and eigen-decomposing to obtain $V_{J-k}$ as same as step (1). Third, the complexity of eigen-decomposing $T_{U}T_{V-k}$ to calculate the *k*-th jamming SV ${\hat{a}}_{k}$ is $O({\left(MN\right)}^{3})$. Suppose that the discrete sampling points of each jamming domain are equal to $P_{0}$, i.e., $P_{k}\;=\;P_{0}$, the complexity of estimating all *K* jamming SVs is $O(K\cdot \left(\max\left({\left(MN\right)}^{2}P_{0},{\left(MN\right)}^{3}\right)\right))$.
3. The complexity of computing ${\hat{w}}_{proposed}$ is $O({\left(MN\right)}^{3})$ owing to matrix inversion of ${\hat{R}}_{j+n}$. Therefore, the overall complexity of the proposed method is roughly $O(K\cdot \left(\max\left({\left(MN\right)}^{2}P_{0},{\left(MN\right)}^{3}\right)\right))$.

## 4. Simulation Results

In this section, the performance of the proposed algorithm against deceptive jamming in FDA-MIMO radar (parameters are listed in Table 1) is verified by experiments. Assume that both jamming 1 and jamming 2 are incident on the array from the mainbeam, and jamming 3 is incident on the array from the side lobe. Table 2 exhibits their actual spatial ranges and angles. In the trials, a maximum unambiguous range is set to contain 250 range gates. Therefore, the desired target is placed at the 200-th range gate, and the three jamming are positioned at the 17-th, 84-th and 150-th range gates, respectively. Furthermore, Table 2 also displays the estimated range and angle between the target and the jamming. The additive noise is modeled as a complex Gaussian zero-mean spatially and temporally white process that has identical variances in each sensor. For the proposed method, the desired target domain and three jamming domains are set as $\Theta_{S}=[(28\;\mathrm{km},36\;\mathrm{km}),(0^{\circ },8^{\circ })]$, $\Theta_{1}=[(38\;\mathrm{km},46\;\mathrm{km}),(0^{\circ },8^{\circ })]$, $\Theta_{2}=[(48\;\mathrm{km},56\;\mathrm{km}),(0^{\circ },8^{\circ })]$ and $\Theta_{3}=[(58\;\mathrm{km},66\;\mathrm{km}),$$(-42^{\circ },-34^{\circ })]$, respectively. All these four range-angle sectors are uniformly sampled to be discrete sectors with the same range interval Δr=0.2km and angle interval $\Delta \theta \;=\;0.2^{\circ }$.

The proposed method is compared to the shrinkage beamformer [21], the eigenspace-based beamformer [24], the IPNCM linear reconstruction-based beamformer (IPNCM-linear) [31], the IPNCM reconstruction beamformer using spatial power spectrum sampling (IPNCM-SPSS) [35], the beamformer in [36], the IPNCM reconstruction beamformer based on signal power estimation (IPNCM-SPE) [39], and the IPNCM reconstruction beamformer using maximum entropy power spectrum (IPNCM-MEPS) [40]. The energy percentage in the Eigenspace beamformer is ρ=0.9. For the INCM-SPSS beamformer, the reference range and angle are set as $\left(r_{0},\theta_{0}\right)=\left(30\;{\mathrm{km},2}^{\circ }\right)$ and the null broadening parameter is $\Delta \;=\;\sin^{-1}\left(2/\left(MN\right)\right)$. The uncertainty level ε=0.5 is set for the beamformer in [36]. The constraint factor in our proposed method satisfies λ=0.95. The Matlab CVX toolbox [44] is applied to solve all optimization problems in the compared methods. When the input SNR is changed, the number of snapshots is fixed to K=50. When we vary the number of snapshots, the input SNR is fixed at 30 dB. In total, 100 Monte-Carlo trials are carried out in each scenario.

### 4.1. Transmit-Receive Beampattern Comparison

**Figure 6:**
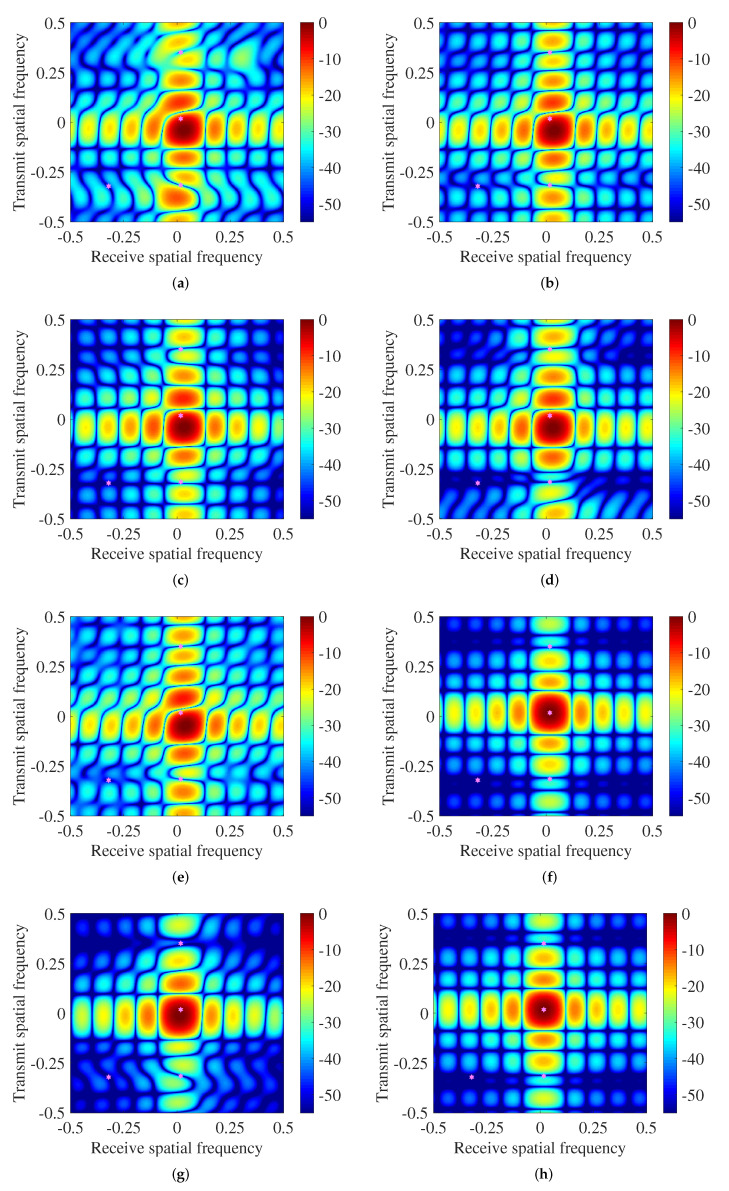
Transmit–receive beamformer of various approaches (SNR = 15 dB): (**a**) Shrinkage. (**b**) Eigenspace. (**c**) IPNCM-linear. (**d**) IPNCM-SPSS. (**e**) Method in [36]. (**f**) IPNCM-SPE. (**g**) IPNCM-MEPS. (**h**) Proposed method.

Figure 6 compares the aforementioned beamforming algorithms in the transmit-receive spatial frequency domain where SNR is 15 dB. The pink hexagonal stars in these diagrams indicate the actual positions of the desired target and jamming. As can be seen, three jamming in all tested algorithms is located at deep notches, indicating that these methods have superior jamming rejection. This is because in Shrinkage and Eigenspace beamformers, the loading factor can be adjusted appropriately according to the characteristics of the received data, always ensuring stronger anti-jamming performance even if the sample data comprise the desired signal, whereas for the IPNCM-linear beamformer, IPNCM-SPSS beamformer, the beamformer in [36], IPNCM-SPE beamformer, IPNCM-MEPS beamformer, and our proposed approach, since they all integrate the Capon spatial spectrum in the range-angle domain that does not contain the desired signal to reconstruct the IPNCM. Therefore, the reconstructed matrix has eliminated the influence of the desired signal and is closer to the theoretical IPNCM, resulting in improved jamming rejection performance. However, as shown in Figure 6a,b,e, the mainbeam of the Shrinkage beamformer, the Eigenspace beamformer, and the beamformer in [36] are not ideal because they require more sample snapshots to guarantee a better main lobe. Moreover, except for the IPNCM-SPE beamformer and proposed beamformer, all tested methods have a mismatch between the mainbeam and the real target SV, which implies that these methods cannot accurately estimate the target SV when the desired signal exists in the training sample, especially under high SNR. Nevertheless, the IPNCM-SPE beamformer and proposed beamformer, as illustrated in Figure 6f,h, outperform the remaining methods in terms of low sidelobe and accurately estimating the desired target SV, revealing that they are able to effectively reduce the influence of training sample contamination and desired target SV mismatch.

### 4.2. Beam Pattern Comparison

**Figure 7:**
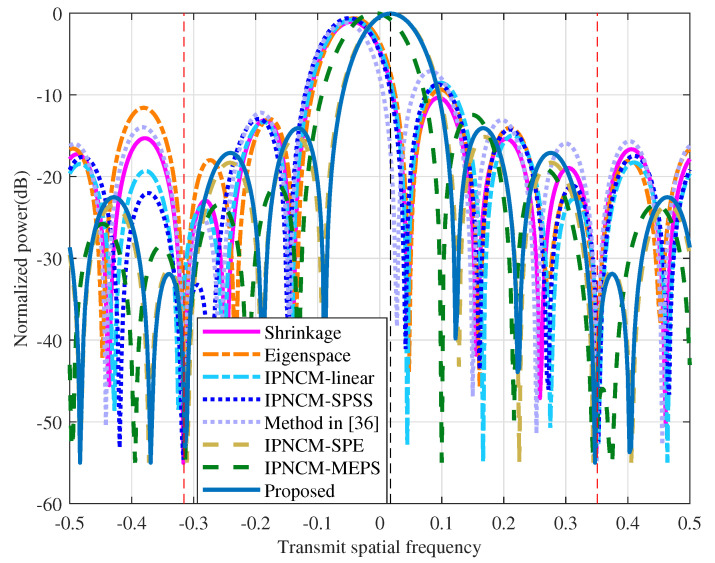
Beam patterns of the tested beamformers in the transmit spatial frequency.

Figure 7 depicts the beam patterns of the proposed algorithm and the tested beamformers in the transmit spatial frequency dimension when the receive spatial frequency is fixed as the mainbeam (the real desired target angle in Table 2 is the mainbeam direction, and the receive spatial frequency can be calculated from Equation (9)). The transmit spatial frequencies of the three can be calculated as 0.0174, −0.3159, and 0.3508, respectively, by Equations (8) and (12), so the black dashed line in Figure 7 corresponds to the desired target transmit spatial frequency position, and the red dashed line represents the mainbeam jamming transmit spatial frequency position. As can be observed in Figure 7, the proposed method is capable of producing a precise mainbeam at the transmit spatial frequency where the desired target is located, resulting in maximum target gain with low side lobes.The performance of the IPNCM-MEPS beamformer is closest to the proposed method, while the mainbeam created by the remaining tested beamformers diverge substantially from the desired target transmit spatial frequency, resulting in target gain attenuation. Furthermore, all tested beamformers can yield notches at the mainbeam jamming, resulting in improved jamming suppression.

This is because the proposed approach and IPNCM-based beamformers depend on the integration of the specific jamming domain to obtain a more accurate IPNCM. The enhanced covariance matrix, rather than the sample covariance matrix, is utilized by the Shrinkage beamformer and Eigenspace beamformer to approximate the theoretical IPNCM. Hence, all of these techniques can augment the robustness to model mismatches.

### 4.3. Beamformer Output Results

**Figure 8:**
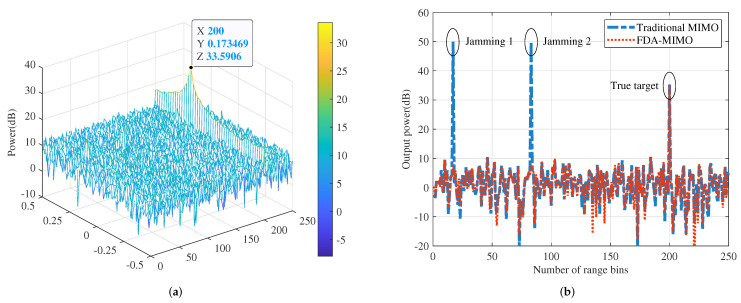
Output results of range-angle two dimensional RAB: (**a**) Range-Doppler filtering output results. (**b**) Output comparison of different radar frameworks.

Figure 8a depicts the filtering output results of the range-Doppler dimension under FDA-MIMO radar. The jamming is effectively inhibited as a consequence of the range dimensional information mismatch, while the desired target situated at the 200-th range gate is retained and achieves the maximum output power. Figure 8b compares the output power profiles of the conventional MIMO radar and FDA-MIMO radar at $\theta \;=\;0^{\circ }$. It can be observed that the transmit SV of a conventional MIMO radar only contains the angle dimension parameter and ignores the DOF in range dimension, hence only the side lobe jamming 3 under angle mismatch can be eliminated, while the range deceptive jamming 1 and jamming 2 in the mainbeam cannot be suppressed. In contrast, FDA-MIMO radar transmit SV incorporates both range and angle information. The proposed RAB approach, which exploits the controlled DOF of the range dimension, can efficiently reject any jamming, including the main lobe direction.

### 4.4. Output SINR Performance

#### 4.4.1. Effect of Residual Noise on Output SINR

**Figure 9:**
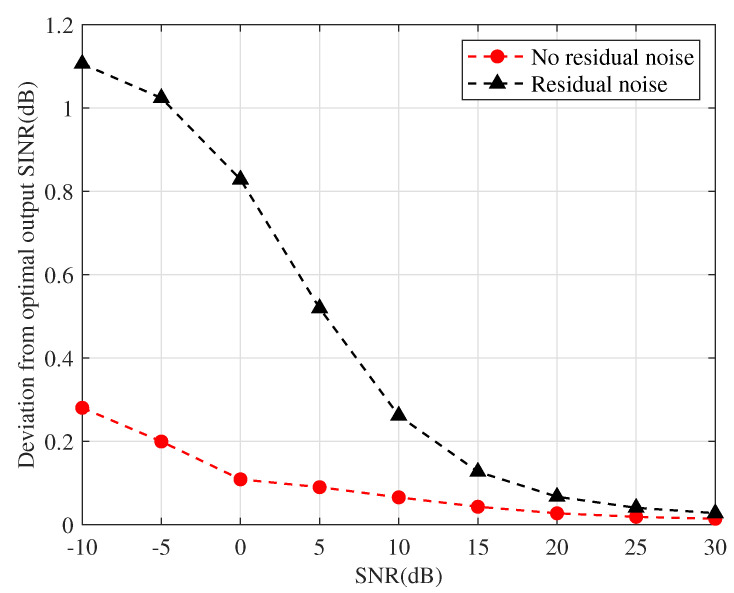
Deviation from optimal SINR versus input SNR.

Figure 9 illustrates the deviation of the output SINR from the optimal SINR with respect to input SNR for both the proposed RAB with and without residual noise rejection. It is clear that the deviation of the proposed approach considering the effect of residual noise is smaller under low SNR, indicating that it is closer to the optimal SINR. Nevertheless, the performance of the algorithm with and without residual noise rejection is essentially the same when the input SNR is higher, which is attributed to the fact that the residual noise is considerably lower than the desired signal power at high SNR, when the influence of both with and without residual noise on the signal covariance matrix reconstruction is negligible, the two algorithms perform equivalently.

#### 4.4.2. Mismatch Due to Signal Look Direction and Range Error

**Figure 10:**
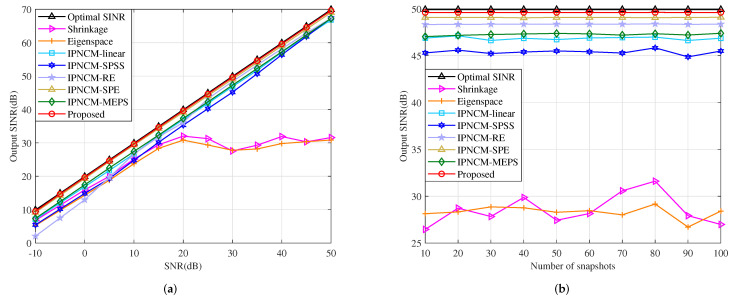
Output SINR in the case of look direction and range error versus (**a**) input SNR, JNR = 30 dB, K = 50. (**b**) the number of snapshots, SNR = 30 dB, JNR = 30 dB.

In this example, the effect of random look direction and range error on beamformer output SINR is demonstrated. The random DOA errors of both the desired signal and the jamming are uniformly distributed in $\left[-3^{\circ },3^{\circ }\right]$, whereas their random range errors are set to be uniformly distributed in [−3km,3km]. This indicates that the actual desired target DOA is uniformly distributed in $\left[1^{\circ },7^{\circ }\right]$ and the range is uniformly distributed in [29km,35km]. The three jamming DOAs, however, are uniformly distributed in $\left[1^{\circ },7^{\circ }\right]$, $\left[1^{\circ },7^{\circ }\right]$ and $\left[-41^{\circ },-35^{\circ }\right]$, where their ranges are specified to obey uniform distribution of [39km,45km], [49km,55km], and [59km,65km]. Note that the angle and range of the desired target, as well as the jamming, vary in each independent trial while maintaining constant among the training samples. Figure 10a plots the output SINR versus input SNR of several tested beamformers. It can be seen that both our proposed method and the IPNCM-SPE beamformer are close to the optimal solution at SNR > 10 dB. Moreover, they have superior performance over the remaining beamformers when −10 dB < SNR < 50 dB. The proposed method and these beamformers based on interference plus noise covariance matrix reconstruction generally outperform the Shrinkage and Eigenspace beamformers at high SNR due to the fact that the interference covariance matrix of the two techniques still contains the desired signal, which can severely attenuate the useful signal output power at high SNR, leading to a decrease in SINR. The curve of output SINR chinging with the number of input snapshots is exhibited in Figure 10b. It is evident that the proposed beamformer basically attain the optimal value and converge substantially faster than the other approaches under low and high snapshot numbers.

#### 4.4.3. Mismatch Due to Array Geometry Error

**Figure 11:**
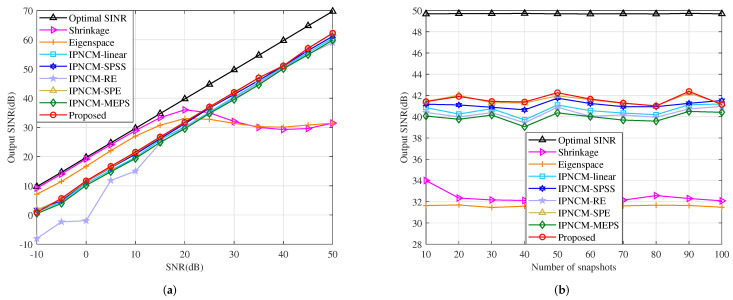
Output SINR in the case of array geometry error versus (**a**) input SNR, JNR = 30 dB, K = 50. (**b**) the number of snapshots, SNR = 30 dB, JNR = 30 dB.

This example is executed in the scenario of SV mismatch owing to array geometry error, where the displacement of each sensor is adjusted far from its theoretical position to obey a uniform distribution within the interval [−0.05,0.05] measured in wavelength. Figure 11a illustrates the variation of the output SINR with respect to input SNR. The output SINR of the proposed beamformer, IPNCM-SPE beamformer and IPNCM-SPSS beamformer, is relatively close to and somewhat superior than that of IPNCM-linear beamformer, beamformer in [36] and IPNCM-MEPS beamformer when SNR >−10 dB. Over a wide range of input SNRs, the Shrinkage beamformer and Eigenspace beamformer exhibit significantly lower output SINRs than these IPNCM-based beamformers. Figure 11b exemplifies the output SINR versus the number of snapshots. We can observe that the snapshot number has no perceptible influence on the IPNCM-based beamformer under the array geometry error condition, and the proposed beamformer typically outperforms the remaining beamformers, whereas the Shrinkage beamformer and Eigenspace beamformer have the worst convergence.

#### 4.4.4. Mismatch Due to Channel Gain and Phase Error

**Figure 12:**
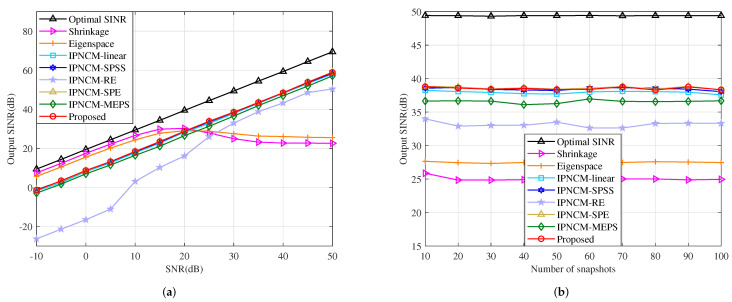
Output SINR in the case of gain and phase error versus (**a**) input SNR, JNR = 30 dB, K = 50. (**b**) the number of snapshots, SNR = 30 dB, JNR = 30 dB.

This example is conducted in the scenario where channel random gain and phase error affect output SINR. Assume that the gain and phase error of each sensor are drawn from random generators $N(1,0.1^{2})$ and $N(0,{\left(2^{\circ }\right)}^{2})$, respectively. The output SINR versus input SNR of tested beamformers is evaluated in Figure 12a. Clearly, the curves of the proposed beamformer, IPNCM-SPE beamformer, IPNCM-SPSS beamformer and IPNCM-linear beamformer are adjacent and preferable than the method in [36]. It can be deduced that the beamformer in [36] has a significant drawback in coping with channel gain and phase error when SNR < 20 dB. Figure 12b demonstrates the output SINR of the tested algorithm versus the number of snapshots. It is straightforward to see that the proposed method, IPNCM-SPE beamformer and IPNCM-SPSS beamformer have the most remarkable properties. Nevertheless, the output SINR of all beamformers is far below the optimal value.

#### 4.4.5. Mismatch Due to Incoherent Local Scattering

In this example, the effect of incoherent local scattering on output SINR is considered. Assume that the desired signal has a time-varying spatial signature that differ from snapshot to snapshot, and its steering vector is modeled as

(47) $$\begin{array}{c} a\left(k\right)=s_{0}\left(k\right)a\left(r_{s},\theta_{s}\right)+\sum_{p=1}^{4}s_{p}\left(k\right)a\left(r_{p},\theta_{p}\right) \end{array}$$

where $a(r_{s},\theta_{s})$ indicates the direct path of the real desired signal location $\left(r_{s},\theta_{s}\right)$, whereas $a(r_{p},\theta_{p}),p=1,2,3,4$ stands for the SV of the incoherent scattering paths. $s_{p}\left(k\right)\left(p=0,1,2,3,4\right)$ are i.i.d. random variable obeying a zero-mean complex Gaussian distribution drawn from the random generator $N(0,1^{2})$. The range $r_{p}\left(p=1,2,3,4\right)$ and the angle $\theta_{p}\left(p=1,2,3,4\right)$ are independently drawn in each trial from the Gaussian random generators $N(2\;\mathrm{km},(2\;{\mathrm{km})}^{2})$ and $N(2^{\circ },{\left(2^{\circ }\right)}^{2})$, respectively. Note that $r_{p}$ and $\theta_{p}$ vary from trial to trial while remaining fixed over snapshots. However, $s_{p}\left(k\right)$ holds changing both trial-to-trial and snapshot-to-snapshot. (47) corresponds to the case of incoherent local scattering [45], where the signal covariance matrix is no longer a rank-one matrix and (16) should be written in a more general form as follows:

(48) $$\begin{array}{c} \mathrm{SINR}=\frac{w^{H}R_{s}w}{w^{H}R_{j+n}w} \end{array}$$

**Figure 13:**
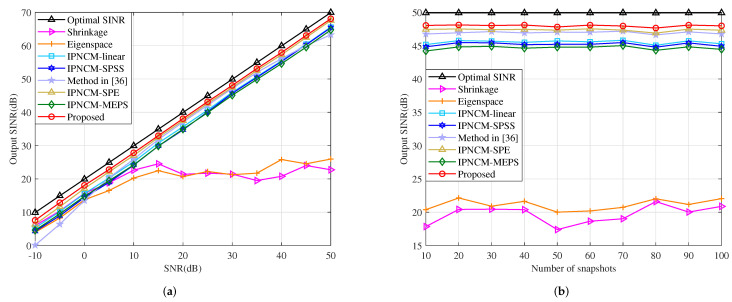
Output SINR in the case of incoherent local scattering versus (**a**) input SNR, JNR = 30 dB, K = 50. (**b**) the number of snapshots, SNR = 30 dB, JNR = 30 dB.

The optimal beamformer weight vector w after maximizing (48) can be obtained from the principal eigenvector of $R_{j+n}^{-1}R_{s}$ [46]. Figure 13a,b depict the output SINR of the tested beamformers versus the input SNR and versus the number of snapshots, respectively. It can be deduced that the proposed beamformer provides a more satisfactory performance in a large SNR interval as well as in the case of small samples, and its output SINR is higher than that of the remaining beamformers, which reveals that the proposed method has extremely robustness for the incoherent local scattering problem of the signal. Furthermore, the IPNCM-linear beamformer, IPNCM-SPSS beamformer and IPNCM-MEPS beamformer perform comparably and significantly worse than the other IPNCM-based beamformers, owing to the fact that they reconstruct the IPNCM in such a way that the integration interval of the interference is the entire remaining range that does not comprise the desired target domain, which increases redundant information resulting in a performance degradation.

#### 4.4.6. Mismatch Due to Coherent Local Scattering

**Figure 14:**
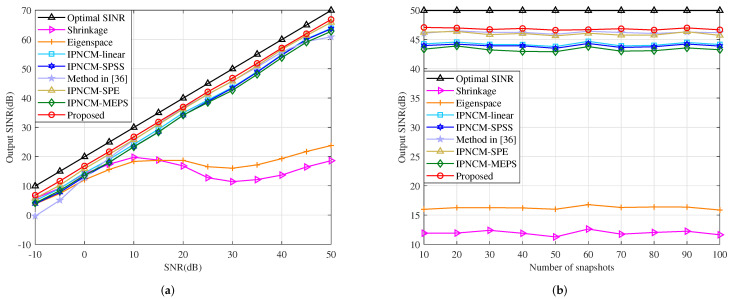
Output SINR in the case of coherent local scattering versus (**a**) input SNR, JNR = 30 dB, K = 50. (**b**) the number of snapshots, SNR = 30 dB, JNR = 30 dB.

In this example, the effect of the desired target SV mismatch due to coherent local scattering on the output SINR is explored. Assume that the real desired target SV is comprised of five coherent signal paths as

(49) $$\begin{array}{c} a\left(k\right)=a\left(r_{s},\theta_{s}\right)+\sum_{p=1}^{4}e^{j\phi_{p}}a\left(r_{p},\theta_{p}\right) \end{array}$$

where $a(r_{s},\theta_{s})$ represents the direct path of the real desired signal location $\left(r_{s},\theta_{s}\right)$, whereas $a(r_{p},\theta_{p}),p=1,2,3,4$ corresponds to the SV of the coherent scattering paths. The range $r_{p}\left(p=1,2,3,4\right)$ and the angle $\theta_{p}\left(p=1,2,3,4\right)$ are independently and uniformly drawn from the interval [24km,36km] and $\left[-4^{\circ },8^{\circ }\right]$ in each trial, respectively. $e^{j\phi_{p}}$ is independently uniformly distributed in the interval [0,2π] in each trial. Note that $r_{p}$, $\theta_{p}$ and $e^{j\phi_{p}}$ vary from trial to trial while remaining fixed over snapshots. Figure 14a exhibits the output SINR versus input SNR. It can be clearly seen that the proposed algorithm has the optimal output SINR compared to the rest of the tested algorithms when the SNR is in the interval, which indicates that the proposed method can effectively cope with the model mismatch under coherent local scattering. The output SINR versus the number of snapshots is plotted in Figure 14b. It is clear that the SINR obtained by the proposed method is closer to the best SINR at different numbers of snapshots and has the fastest convergence rate.

#### 4.4.7. Mismatch Due to SV Random Error

**Figure 15:**
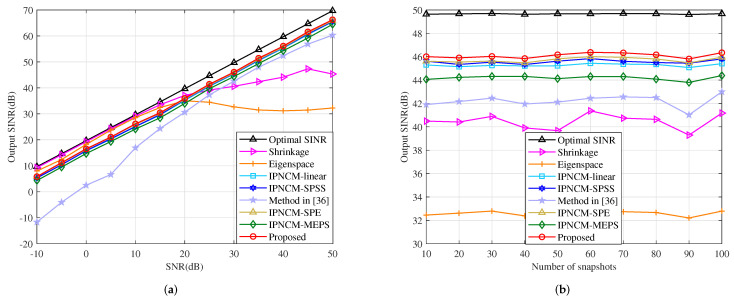
Output SINR in the case of SV random error versus (**a**) input SNR, JNR = 30 dB, K = 50. (**b**) the number of snapshots, SNR = 30 dB, JNR = 30 dB.

In this example, the effect of SV random error on output SINR is investigated. Assume the real desired target and jamming SVs are composed of the corresponding presumed SVs superimposed with a random error vector, modeled as follows

(50) $$\begin{array}{c} a_{l}=\tilde{a}+e_{l},l=0,1,2,\cdots L \end{array}$$

where $e_{l}$ indicates the random error vector, which can be written as

(51) $$\begin{array}{c} e_{l}=\frac{\chi_{l}}{\sqrt{MN}}{\left[e^{j\varphi_{0}^{l}},e^{j\varphi_{1}^{l}},\cdots ,e^{j\varphi_{MN-1}^{l}}\right]}^{T} \end{array}$$

where $\chi_{l}$ represents the Euclidean norm of $e_{l}$ and is uniformly distributed in the interval [0,6]. $\varphi_{i}^{l},i=0,1,\cdots ,MN-1$ signifies the phase of the random vector error, which obeys a uniform distribution in the interval [0,2π]. The SV random error model established in (50) can be interpreted as a result of numerous error factors including look direction error, array calibration error and so on. The relationship between the output SINR and input SNR of the tested beamformers is described in Figure 15a. When −10 dB < SNR < 50 dB, the proposed method has the highest output SINR among the IPNCM-based beamformers, and also outperforms the Shrinkage beamformer and Eigenspace beamformer if SNR > 20 dB. This is because at high SINR, the desired signal in the training sample has a significant influence on the sample covariance matrix, whereas the propose method and other the IPNCM-based beamformers construct the IPNCM by integrating the region where the jamming domain is situated, which removes the effect of the desired signal to a degree, resulting in excellent performance under high SNR conditions. When SNR < 20 dB, the Shrinkage beamformer and Eigenspace beamformer outperform the IPNCM-based beamformers because the former two employ the enhanced covariance matrix instead of the sample covariance matrix, which guarantees the robustness against SV random error. However, these IPNCM-based beamformers presume that the antenna array has no calibration errors (for example, the channel gain and phase error, and array geometric error). As previously stated, the SV random error contains numerous error factors, including the array calibration error, which causes the IPNCM-based beamformers cannot handle the SV random error adequately under low SNR. These analyses are also consistent with the simulation results for array geometry error, gain and phase error illustrated in Figure 11 and Figure 12.

Figure 15b depicts the output SINR versus the number of snapshots of the tested beamformers. As can be demonstrated, the proposed method has a faster convergence rate and a higher output SINR than other beamformers.

## 5. Conclusions

In this paper, a novel RAB algorithm based on the desired target SV estimation and IPNCM reconstruction is introduced to improve the performance of FDA-MIMO radars in suppressing mainbeam deceptive jamming. In this approach, we utilize a modified Capon power spectrum with residual noise eliminated to estimate the desired target SV, and a new method is devised to construct jamming covariance matrix based on jamming SV and power estimation. In terms of preventing mainbeam deceptive jamming and coping with scenarios when the SV is mismatched and the desired signal contaminates the training samples, the proposed beamformer is more effective than several existing RAB technologies. Moreover, it simply requires a priori knowledge of the range-angle domain in which the desired target and jamming may exist. Simulation results have demonstrated that the proposed RAB approache outperforms some existing RAB methods in instances of look direction and range error, channel gain and phase error, array geometry error, incoherent local scattering, coherent local scattering and SV random error.

## Figures and Tables

**Table 1:** FDA-MIMO radar parameters.

Parameter	Value	Parameter	Value
M	10	N	10
Element spacing	0.15 m	Carrier frequency	1 GHz
Frequency increment	5 kHz	PRF	4 kHz
Bandwidth	1 MHz	Number of pulses	200

**Table 2:** Desired target and deceptive jamming parameters.

Parameter	Desired Target	Jamming 1	Jamming 2	Jamming 3
SNR/INR	15 dB	30 dB	30 dB	30 dB
Real range	30 km	40 km	50 km	60 km
Real angle	$2^{\circ }$	$2^{\circ }$	$2^{\circ }$	$-40^{\circ }$
Presumed range	32 km	42 km	52 km	62 km
Presumed angle	$4^{\circ }$	$4^{\circ }$	$4^{\circ }$	$-38^{\circ }$

## Data Availability

Not applicable.

## References

[1] Tan M., Wang C., Xue B., Xu J. (2021). A novel deceptive jamming approach against frequency diverse array radar. IEEE Sens. J..

[2] Zhang S., Zhou Y., Zhang L., Zhang Q., Du L. (2021). Target Detection for Multistatic Radar in the Presence of Deception Jamming. IEEE Sens. J..

[3] Zhou C., Liu Q., Chen X. (2018). Parameter estimation and suppression for DRFM-based interrupted sampling repeater jammer. IEEE Sens. J..

[4] Lan L., Liao G., Xu J., Zhang Y., Zhu S. (2021). Mainlobe deceptive jammer suppression using element-pulse coding with MIMO radar. Signal Process..

[5] Lan L., Liao G., Xu J., Zhang Y., Liao B. (2020). Transceive beamforming with accurate nulling in FDA-MIMO radar for imaging. IEEE Trans. Geosci. Remote Sens..

[6] Xu J., Liao G., Zhu S., So H.C. (2015). Deceptive jamming suppression with frequency diverse MIMO radar. Signal Process..

[7] Li S., Zhang L., Liu N., Zhang J., Zhao S. (2017). Adaptive detection with conic rejection to suppress deceptive jamming for frequency diverse MIMO radar. Digit Signal Process..

[8] Lan L., Liao G., Xu J., Zhang Y., Fioranelli F. (2018). Suppression approach to main-beam deceptive jamming in FDA-MIMO radar using nonhomogeneous sample detection. Digit Signal Process..

[9] Xu J., Kang J., Liao G., So H.C. Mainlobe deceptive jammer suppression with FDA-MIMO radar. Proceedings of the 2018 IEEE 10th Sensor Array and Multichannel Signal Processing Workshop (SAM).

[10] Liu Q., Xu J., Ding Z., So H.C. (2020). Target localization with jammer removal using frequency diverse array. IEEE Trans. Veh. Technol..

[11] Wang Y., Zhu S. (2020). Main-beam range deceptive jamming suppression with simulated annealing FDA-MIMO radar. IEEE Sens. J..

[12] Lan L., Xu J., Liao G., Zhang Y., Fioranelli F., So H.C. (2020). Suppression of mainbeam deceptive jammer with FDA-MIMO radar. IEEE Trans. Veh. Technol..

[13] Ge J., Xie J., Wang B. (2021). A cognitive active anti-jamming method based on frequency diverse array radar phase center. Digit Signal Process..

[14] Zhang X., He Z., Liao B., Zhang X., Peng W. (2018). Robust quasi-adaptive beamforming against direction-of-arrival mismatch. IEEE Trans. Aerosp. Electron. Syst..

[15] Chen P., Yang Y., Wang Y., Ma Y. (2018). Robust adaptive beamforming with sensor position errors using weighted subspace fitting-based covariance matrix reconstruction. Sensors.

[16] Yao D., Zhang X., Hu B., Yang Q., Wu X. (2020). Robust Adaptive Beamforming with Optimal Covariance Matrix Estimation in the Presence of Gain-Phase Errors. Sensors.

[17] Zhuang J., Xue Y., Kang J., Chen D., Wan Q. (2021). Robust adaptive beamforming under data dependent constraints. Signal Process..

[18] Li J., Stoica P., Wang Z. (2003). On robust Capon beamforming and diagonal loading. IEEE Trans. Aerosp. Electron. Syst..

[19] Elnashar A., Elnoubi S.M., El-Mikati H.A. (2006). Further study on robust adaptive beamforming with optimum diagonal loading. IEEE Trans. Aerosp. Electron. Syst..

[20] Yang J., Ma X., Hou C., Liu Y. (2009). Automatic generalized loading for robust adaptive beamforming. IEEE Signal Process. Lett..

[21] Du L., Li J., Stoica P. (2010). Fully automatic computation of diagonal loading levels for robust adaptive beamforming. IEEE Trans. Aerosp. Electron. Syst..

[22] Huang F., Sheng W., Ma X. (2012). Modified projection approach for robust adaptive array beamforming. IEEE Trans. Aerosp. Electron. Syst..

[23] Zhou M., Ma X., Shen P., Sheng W. (2019). Weighted subspace-constrained adaptive beamforming for sidelobe control. IEEE Commun. Lett..

[24] Jia W., Jin W., Zhou S., Yao M. (2013). Robust adaptive beamforming based on a new steering vector estimation algorithm. Signal Process..

[25] Vorobyov S.A., Gershman A.B., Luo Z.Q. (2003). Robust adaptive beamforming using worst-case performance optimization: A solution to the signal mismatch problem. IEEE Trans. Signal Process..

[26] Liao B., Guo C., Huang L., Li Q., So H.C. (2003). Robust adaptive beamforming with precise main beam control. IEEE Trans. Aerosp. Electron. Syst..

[27] Zhuang J., Shi B., Zuo X., Ali A.H. (2003). Robust adaptive beamforming with minimum sensitivity to correlated random errors. Signal Process..

[28] Beck A., Eldar Y.C. (2007). Doubly constrained robust Capon beamformer with ellipsoidal uncertainty sets. IEEE Trans. Signal Process..

[29] Vorobyov S.A., Chen H., Gershman A.B. (2008). On the relationship between robust minimum variance beamformers with probabilistic and worst-case distortionless response constraints. IEEE Trans. Signal Process..

[30] Jiang X., Zeng W.J., Yasotharan A., So H.C. (2014). Robust beamforming by linear programming. IEEE Trans. Signal Process..

[31] Gu Y., Leshem A. (2012). Robust adaptive beamforming based on interference covariance matrix reconstruction and steering vector estimation. IEEE Trans. Signal Process..

[32] Lu Z., Li Y., Gao M., Zhang Y. (2013). Interference covariance matrix reconstruction via steering vectors estimation for robust adaptive beamforming. Electron. Lett..

[33] Huang L., Zhang J., Xu X., Ye Z. (2015). Robust adaptive beamforming with a novel interference-plus-noise covariance matrix reconstruction method. Electron. Lett..

[34] Shen F., Chen F., Song J. (2015). Robust adaptive beamforming based on steering vector estimation and covariance matrix reconstruction. IEEE Commun. Lett..

[35] Zhang Z., Liu W., Leng W., Wang A., Shi H. (2016). Interference-plus-noise covariance matrix reconstruction via spatial power spectrum sampling for robust adaptive beamforming. IEEE Signal Process Lett..

[36] Zhang Y., Li Y., Gao M. (2016). Robust adaptive beamforming based on the effectiveness of reconstruction. Signal Process..

[37] Liu F., Du R., Wu J., Zhou Q., Zhang Z., Cheng Y. (2016). Multiple Constrained *l*_2_-Norm Minimization Algorithm for Adaptive Beamforming. IEEE Signal Process Lett..

[38] Zheng Z., Yang T., Wang W.Q., So H.C. (2019). Robust adaptive beamforming via simplified interference power estimation. IEEE Trans. Aerosp. Electron. Syst..

[39] Zheng Z., Wang W.Q., So H.C., Liao Y. (2019). Robust adaptive beamforming using a novel signal power estimation algorithm. Digit Signal Process..

[40] Mohammadzadeh S., Nascimento V.H., de Lamare R.C., Kukrer O. (2020). Maximum entropy-based interference-plus-noise covariance matrix reconstruction for robust adaptive beamforming. IEEE Trans. Aerosp. Electron. Syst..

[41] Zhang P., Yang Z., Liao G., Jing G., Ma T. (2021). An RCB-Like Steering Vector Estimation Method Based on Interference Matrix Reduction. IEEE Trans. Aerosp. Electron Syst..

[42] Zhuang J., Manikas A. (2013). Interference cancellation beamforming robust to pointing errors. IET Signal Process..

[43] Zhang F., Zhang Q. (2006). Eigenvalue inequalities for matrix product. IEEE Trans. Automat. Contr..

[44] Grant M., Boyd S. (2020). CVX: Matlab Software for Disciplined Convex Programming, Version 2.2. http://cvxr.com/cvx.

[45] Besson O., Stoica P. (2000). Decoupled estimation of DOA and angular spread for a spatially distributed source. IEEE Trans. Signal Process..

[46] Gershman A.B. (1999). Robust adaptive beamforming in sensor arrays. Int. J. Electron. Commun..

